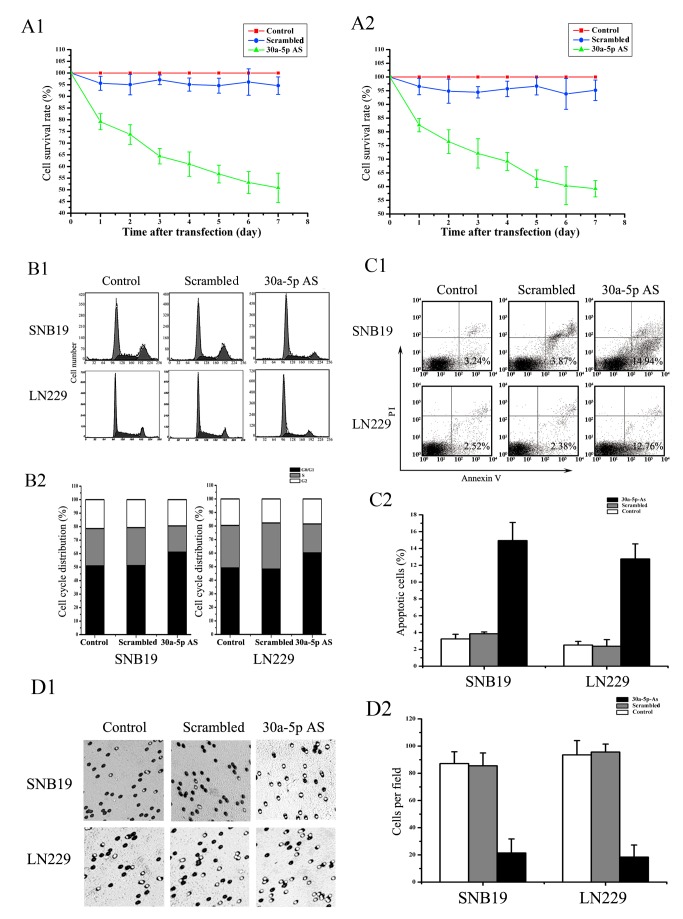# Correction: MiR-30a-5p Antisense Oligonucleotide Suppresses Glioma Cell Growth by Targeting SEPT7

**DOI:** 10.1371/annotation/9a8447a4-bffe-445f-b323-7e200896aea9

**Published:** 2013-10-10

**Authors:** Zhifan Jia, Kun Wang, Guangxiu Wang, Anling Zhang, Peiyu Pu

The published Figure 2 is incorrect. Please see the corrected Figure 2 here: 

**Figure:**